# Evolved genetic and phenotypic differences due to mitochondrial-nuclear interactions

**DOI:** 10.1371/journal.pgen.1006517

**Published:** 2017-03-31

**Authors:** Tara Z. Baris, Dominique N. Wagner, David I. Dayan, Xiao Du, Pierre U. Blier, Nicolas Pichaud, Marjorie F. Oleksiak, Douglas L. Crawford

**Affiliations:** 1 Marine Biology and Ecology, Rosenstiel School of Marine and Atmospheric Sciences, University of Miami, Rickenbacker Causeway, Miami, FL, United States of America; 2 Dept de Biologie, Université du Québec à Rimouski, 300 Allée des Ursulines, Rimouski, Quebec, Canada; Fred Hutchinson Cancer Research Center, UNITED STATES

## Abstract

The oxidative phosphorylation (OxPhos) pathway is responsible for most aerobic ATP production and is the only pathway with both nuclear and mitochondrial encoded proteins. The importance of the interactions between these two genomes has recently received more attention because of their potential evolutionary effects and how they may affect human health and disease. In many different organisms, healthy nuclear and mitochondrial genome hybrids between species or among distant populations within a species affect fitness and OxPhos functions. However, what is less understood is whether these interactions impact individuals within a single natural population. The significance of this impact depends on the strength of selection for mito-nuclear interactions. We examined whether mito-nuclear interactions alter allele frequencies for ~11,000 nuclear SNPs within a single, natural *Fundulus heteroclitus* population containing two divergent mitochondrial haplotypes (mt-haplotypes). Between the two mt-haplotypes, there are significant nuclear allele frequency differences for 349 SNPs with a p-value of 1% (236 with 10% FDR). Unlike the rest of the genome, these 349 outlier SNPs form two groups associated with each mt-haplotype, with a minority of individuals having mixed ancestry. We use this mixed ancestry in combination with mt-haplotype as a polygenic factor to explain a significant fraction of the individual OxPhos variation. These data suggest that mito-nuclear interactions affect cardiac OxPhos function. The 349 outlier SNPs occur in genes involved in regulating metabolic processes but are not directly associated with the 79 nuclear OxPhos proteins. Therefore, we postulate that the evolution of mito-nuclear interactions affects OxPhos function by acting upstream of OxPhos.

## Author summary

Maintaining two distinct mitochondrial haplotypes with functionally important nucleotide variations in a single population is difficult. If interactions with the mitochondria affect the nuclear genome such that some alleles are more advantageous when they occur with a specific mitochondrial genome, then there is an additional genetic burden that also should favor a single mitochondrion. Yet in a single, well mixed *Fundulus heteroclitus* (teleost fish) population where individuals appear to freely interbreed, there are two mitochondria with five amino acid substitutions between them. We investigate this population and show that individuals with different mitochondria have several hundred loci with extreme allele frequency biases. These differences are not due to neutral or demographic processes and affect cardiac energetics. We conclude that the allele frequency differences are most likely evolving by natural selection and affecting ATP production and energy homeostasis.

## Introduction

The Oxidative Phosphorylation (OxPhos) pathway is composed of approximately 89 proteins encoded by the two genomes in animal cells: all 13 mitochondrial proteins and 76 nuclear proteins. These proteins form the five OxPhos enzyme complexes and are responsible for most cellular ATP production. Genetic defects in the OxPhos proteins affect at least 1 in 8,000 people and are the cause for the most common inherited metabolic diseases [1–3]. The interactions between mitochondrial and nuclear OxPhos proteins may be equally as important as deleterious mutations within either genome. The importance of these interactions have been demonstrated experimentally in humans, *Mus*, *Drosophila*, *Tigriopus*, *Callosobruchus*, and *Saccharomyce* where healthy nuclear and mitochondrial genome hybrids between species or among distant populations within a species affect fitness and OxPhos functions *[4–19].* For example, hybrid breakdown due to mito-nuclear incompatibilities among *Tigriopus californicus* populations occur in F2 individuals [7] and alter ROS production [6], OxPhos enzyme activities [20], ATP production and survival [21]. These mito-nuclear interactions (GxG) are often affected by the environment as demonstrated by *Drosophila simulans* mitochondria that have pleiotropic effects at high environmental temperatures when substituted into one *D*. *melanogaster* genotype but not another [11, 22, 23]. Similarly, in seed beetles, *Callosobruchus maculatus*, temperature dependent metabolic rates rely on the interactions between the mitochondrial and nuclear genomes [24]. These mito-nuclear interactions that affect OxPhos are biologically important because they affect fitness (egg production, survivorship, and mating success) [5, 7, 10, 11, 21–23, 25, 26]. In general, these data suggest that mito-nuclear interactions among species or divergent populations are likely to affect an organism’s physiology and these interactions are environmentally dependent [11, 26–29].

Mito-nuclear interactions between different species or populations affect biological function [4–19]. However, it is less understood whether these interactions impact individuals within a single natural population. Theoretically, natural selection due to mito-nuclear interactions could alter allele frequencies when one mt-haplotype has greater fitness with a specific nuclear allele [30]. To determine if mito-nuclear interactions affect genotypes in naturally occurring populations, we examined a *Fundulus heteroclitus* population from a single inter-tidal estuarine creek. This population, just south of the Hudson River in Mantoloking NJ, USA, has two major mt-haplotypes with five non-synonymous substitutions: a “northern” haplotype, common in populations north of the Hudson River and a “southern” haplotype, common in populations south of the Hudson River [31]. The genetic divergence among mt-haplotypes may have been influenced by a historical break at the Hudson River due to the last glaciation [32], enhancing nucleotide pattern differences between northern and southern populations [33]. Populations with two distinct mt-haplotypes are the result of secondary-intergradation, whereby migrants meet where there was once a physical barrier [34]. Importantly, *F*. *heteroclitus* has large populations with low migration rates and is adapted to local environmental conditions [35–41]. Northern populations experience temperatures more than 12°C colder than southern populations and have evolved adaptations to temperature in cardiac metabolism and enzyme expression [39, 42]. Because the evolutionary variation in OxPhos genes has been associated with divergence among populations in response to environmental variation [30, 43–45] and has also been proposed to drive speciation [10, 29, 46–48], we might expect the mt-haplotype to affect the nuclear genotype and alter OxPhos function in the admixed population [49].

To explore potentially evolved epistatic interactions between nuclear and mitochondrial genomes, we addressed two questions: are allele frequencies at nuclear loci significantly different between the two specific mt-haplotypes, and if so, do these differences affect OxPhos function? To answer these questions, 155 Mantoloking, NJ *F*. *heteroclitus* individuals were genotyped at >11,000 SNPs, and their cardiac OxPhos metabolisms were measured. Individuals with southern and northern mt-haplotypes are present at a 60/40 ratio, respectively. We demonstrate significant allele frequency differences at 349 SNP loci between the two mt-haplotypes, and the different nuclear genotype and mt-haplotype combinations are associated with significant OxPhos metabolic differences.

## Results and discussion

Two genotyping by sequencing (GBS) [50] datasets were used: 1) MK-specific (individuals from a single Mantoloking, NJ population) to assess nuclear-mitochondrial associations, and 2) 3-population dataset (Maine, MK, and Georgia individuals) to ascertain the effect of recent admixture. After filtering, 11,705 nuclear SNPs were distributed among 10,180 *F*. *heteroclitus* genome-scaffolds [51] for the MK-specific dataset, and 10,471 for the 3-population dataset. All SNPs and annotations were derived from the 64bp sequence tags used to call SNPs.

### Nuclear-mitochondrial associations

**Fig 1:**
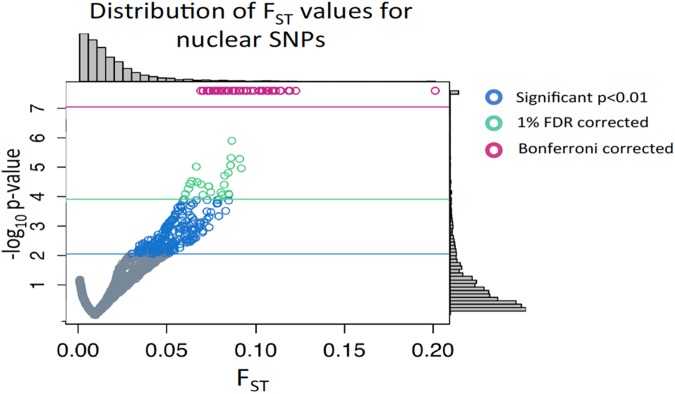
Distribution of *w*F_ST_ values for nuclear SNPs calculated between the two mt-haplotypes within the single MK population. Although we are examining a single population, we use the two mt-haplotypes as artificial populations for *w*F_ST_ calculations. Plot contains *w*F_ST_ values and corresponding negative log_10_ p-values -log_10_(0.01) = 2). Blue values are significant with a p-value <0.01, green values are significant with a 1% FDR correction, and purple values are significant with a Bonferroni correction. Histograms show *w*F_ST_ and p-value distributions.

Strong selection at many nuclear loci creates a genetic load that is detrimental to a species' survival [52, 53]. Therefore, it is unlikely that a population could maintain biologically important mito-nuclear interactions at many loci in a panmictic population (where migration = 0.5 of effective population size, Ne) [54–56]. We suggest that selection due to mito-nuclear interactions may occur if there is extensive standing genetic variation and many genes of small effect affect biological traits. To investigate whether these interactions between genomes do affect allele frequencies, we calculated F_ST_ values for each of the 11,705 SNPs in the MK-specific dataset, using the two mt-haplotypes as independent groups or populations. F_ST_ provides a statistically robust measure of the relative allelic variation between groups *versus* within groups. We denote this within population value as *w*F_ST_. To be clear, although we are examining a single population, we use the two mt-haplotypes as artificial populations for *w*F_ST_ calculations. We found that 349 nuclear SNPs have *w*F_ST_ values that are large statistical outliers (p<0.01; Fig 1). Supplemental tables (S1 and S2 Tables) provide information on genome location, read depth, *w*F_ST_ values, p-values and allele frequencies for all 11,706 SNPs, and location, annotation and 64bp tag sequence for each of the 349 outlier SNPs.

SNPs with *w*F_ST_ outliers values are defined as having significantly large *w*F_ST_ values that are unlikely to occur relative to SNPs with similar heterozygosity (He) [57–59]. With 10% or 1% FDR, 236 or 72, respectively, of these 349 nuclear SNPs were significant; with a more conservative Bonferroni’s correction, 44 SNPs were significant (Fig 1). Among the 349 outlier SNPs, none had significant linkage disequilibrium with each other (D’ is not significant, p > 0.1 and r^2^ <0.3). Differences in minor allele frequency (MiAF) could affect *w*F_ST_ values [60], yet outlier *versus* non-outlier SNPs have similar MiAF: mean MiAF = 0.132 and 0.162 for 9,440 non-outlier, non-significant SNPs (this excludes SNPs that had significant F_ST_ values but not significant outliers) and 349 outlier SNPs respectively (S1 Fig). A separate analysis using allele counts for a Fisher Exact test revealed 229 SNPs with significantly biased allele frequencies (p<0.01). Of these 229 SNPs, 219 were also *w*F_ST_ outliers.

To investigate whether dividing individuals into two arbitrary groups can produce many significant *w*F_ST_ values, we produced a thousand random permutations for 9,440 non-significant SNPs (S2 Fig). None of the 1,000 permutations across 9,440 SNPs produces many *w*F_ST_ values as large as the 349 outlier *w*F_ST_ values as seen in the small overlap in their distributions (S2 Fig). Furthermore, the 99% upper confidence level for the arbitrary *w*F_ST_ values is less than the minimum *w*F_ST_ value for the 349 outlier SNPs (>0.002 and 0.0269, respectively, S2 Fig). Thus, grouping individuals into two arbitrary groups produces few SNPs with significant *w*F_ST_ values, indicating that the 349 outlier SNPs are statistically meaningful.

Each of the 349 outlier SNPs has a *w*F_ST_ value dissimilar from the genome wide *w*F_ST_ value (Fig 1) and is unlikely to occur (S2 Fig). However, even though the 349 outlier *w*F_ST_ values are unlikely, the data could still suffer from type I error. We proceed with our analyses using the 349 outlier SNPs for three reasons. First, to balance type I and type II errors—there are likely to be many more significant SNPs we have not discovered because of the weakness of adaptive tests [59, 61]. Second, the use of different FDR values (1% -10%) yields a large range of significant SNPs (72 to 236), and it has been argued that FDR of 20% or more may be appropriate [62]. Third, and most importantly, we are asking if these 349 outlier SNPs are related to population structure and mitochondrial physiology. Including false positives (type I error) will not bias these tests except to make them less likely to find significant structure or association.

**Fig 2:**
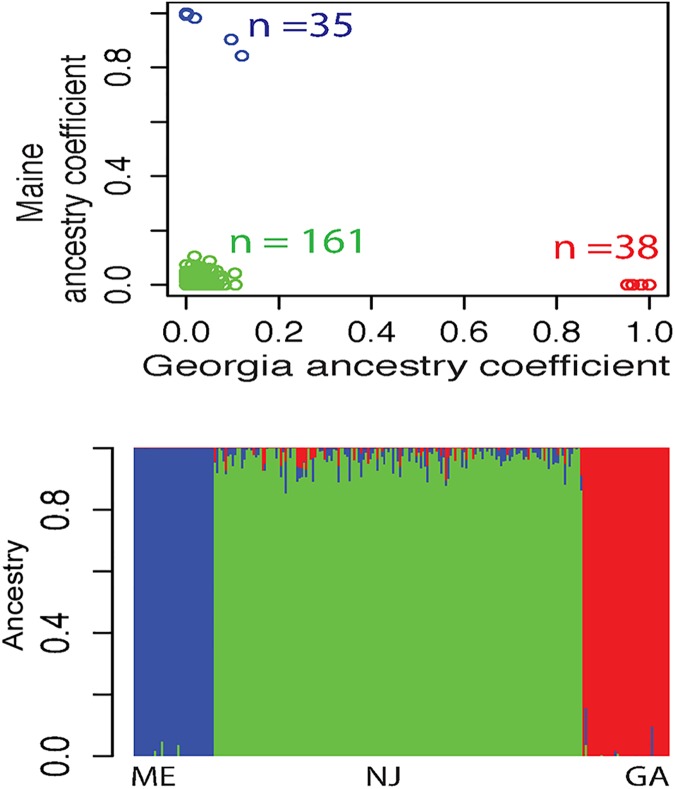
Admixture Analysis on 3-population 3,700 SNP data set. **A:** Plot of Admixture ancestry fractions (Q) from the 3-population SNP data set. 35 Maine individuals are blue, 38 Georgia individuals are red and the 161 MK New Jersey individuals are green. Georgia ancestry coefficients are on the x-axis, and Maine ancestry coefficients are on the y-axis. B: Individual ancestry fractions. Colors are the same as in A. Each individual is represented by a thin vertical line, which is partitioned into 3 colored segments that represent the individual’s estimated membership into one of the three populations.

Given the evolutionary history of *F*. *heteroclitus* and the observation that the MK population has both mt-haplotypes, recent admixture may bias allele frequencies between individuals with northern and southern mt-haplotypes. In order to ascertain the effect of recent admixture, we used Admixture version 1.3.0 to infer ancestries from the 3-population SNP dataset (Fig 2). For the Admixture analysis, we thinned the 3-population 10K SNPs so that all SNPs were >100bp apart, resulting in 3,700 thinned SNPs. Among these 3,700 SNPs, the MK population’s average admixture was 3.2% and never exceeded 14.7%. The plot of ancestry fraction (Q values) from Admixture clearly distinguishes Maine and Georgia from MK (Fig 2B). These analyses indicate that the MK population is a separate and independent population from Maine and Georgia with little recent ancestral admixture and that allele frequency differences between mt-haplotypes within MK are not due to shared genealogies with mt-DNAs. Significant LDs for 3,700 thinned SNPs in the 3-population data are rare and physically close together (S3 Fig): 5 SNP pairs have significant LD (FDR <0.1) with the largest distance = 222 bp. In comparison, nearly all SNP pairs within a scaffold (98%) are > 1,000 bp apart (S4A Fig).

Even though there is little indication of recent admixture (Fig 2), the outlier *w*F_ST_ values could arise with the recent admixing of distinct populations resulting in SNPs with linkage disequilibrium over large distances [63, 64]. Thus we might expect long distance LD for the 349 outlier SNPs with other nuclear SNPs or with mt-haplotypes. Among the 11K MK SNPs are 221,348 calculated LDs using a 50 SNP sliding window; 6,401 of these are significant with 10% FDR [65], and yet only 66 are significant and >100bp apart (S4B Fig). That is, 99% of all SNPs with significant LDs are less than 100bp apart (S4B Fig). This is also true for the 349 outlier SNPs. For each of the 349 outlier SNPs, there are only 229 SNPs in significant LD (FDR 10%) with any other of the 11K SNPs (S4C Fig). Of these 229, six are between SNPs >100bp apart, and among these six, five are less than 200bp apart. One of the 349 outlier SNPs is in LD with another SNP greater than a million bp apart, yet this SNP lacks significant LD with many other closer SNPs. Between each of the 11K SNPs and the mt-haplotype, none are in LD with any reasonable FDR (minimum FDR 0.57). Thus, none of the 349 outlier SNPs are in LD with the mitochondria. The lack of LD between any of the 349 outlier SNPs and the mitochondria reflects the relatively small allele frequency differences between mt-haplotypes for the 349 outliers SNPs (S1 Fig). Specifically, no SNP is close to fixation between mt-haplotypes (*i*.*e*., a difference close to 1). These patterns of LD among nuclear SNPs, including between the 349 outlier SNPs with mitochondrial SNPs, do not support recent admixture. Thus, based on LDs, there is little evidence that the biased allele frequencies in the 349 outlier SNPs are due to recent admixture.

We performed a Tajima’s D analysis on the 11,706 SNPs with a 50bp window using VCFtools [66]. The Tajima’s D value distributions for non-significant and outlier 349 SNPs (S5 Fig) are similar, and the 1% tail of these distributions have equal frequencies of both significant and non-significant SNPs (p-value > 0.2). Tajima’s D compares pair-wises differences to the number of segregating sites where linked SNPs should share large positive values when associated with balancing selection. Our Tajima’s D analysis provides no support for balancing selection. This result likely reflects how SNPs are called: SNPs are called from 64 bp sequenced tags that are typically hundreds of thousands to millions of bps apart (S4A Fig), and only 0.1% of SNPs are in LD with other SNPs (S4B Fig). The limited LD among SNPs within a 64bp tag, and the distance among tags suggest that significant Tajima’s D values are unlikely to occur because the assumption for Tajima’s D analyses is that sites affected by non-neutral processes will affect nearby linked sites. The rarity of SNPs in LD (S4 Fig) and the large MiAF (S1 Fig) suggest that SNPs have existed as long-term standing genetic variation; Tajima’s D analyses are unlikely to detect selection in this case [67].

**Fig 3:**
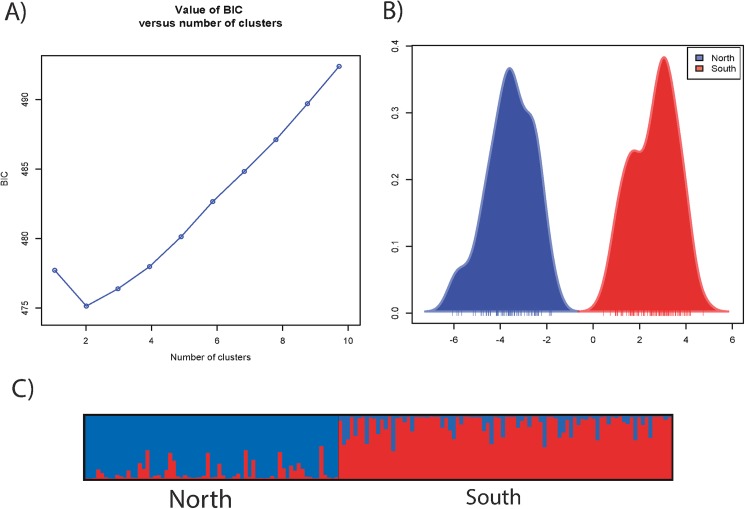
DAPC and STRUCTURE plots. Population structure based on 349 outlier SNPs. **A)** Results of the Bayesian information criterion used to infer the number of genetic clusters when using 349 outlier SNPs. **B)** Discriminant analysis of principal components (DAPC) based on 349 outlier SNPs. Discriminant function separates individuals into two distinct groups, which are the two mt-haplotypes. “North” is for northern mt-haplotype. “South” is for southern mt-haplotype. C) STRUCTURE plot for 349 outlier SNPs. Plot shows probability that individuals’ nuclear genetic variation belongs to one of two clusters, which are northern and southern haplotypes. Each individual is represented by a thin vertical line, which is partitioned into two colored segments that represent the individual’s estimated membership into one or the other cluster. Twenty-one individuals have a mixed ancestry (> 30% of alternate SNP alleles).

To confirm and explore if there is any hidden population structure, we applied discriminant analysis of principal components (DAPC) [68]. Using the 349 outlier SNPs, DAPC identified two groups as the most parsimonious grouping: those associated with the northern or southern mt-haplotype (Fig 3A and 3B). Using all 11K SNPs or 9,440 non-outlier, non significant SNPs, revealed a single grouping (S6 Fig). The observation that distinct groups are not seen with all 11K SNPs (S6 Fig) or the 9,440 non-outlier, non-significant SNPs but are seen with the 349 outlier SNPs lends additional evidence that there is a single well mixed population and the outlier SNPs clearly discriminate individuals into two mt-haplotype associated groups.

STRUCTURE analyses [69] with K = 1–5 using MK’s 349 outlier SNPs corroborated the DAPC results (Fig 3C): K = 2 was much more likely than K = 1 and was the best supported K based on ΔK (the rate of change Ln-likelihood [70, 71]). With K = 2, individuals form two distinct clusters associated with each mt-haplotype. Larger Ks did not produce more definitive groups.

**Fig 4:**
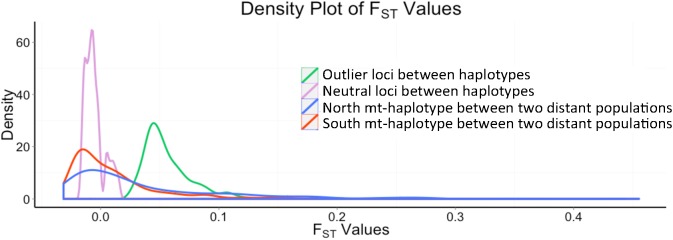
Density plot of F_ST_ values for both between mt-haplotypes within MK population and between populations. Areas under each curve are the same. Green line is for the 349 outlier SNP F_ST_ values between the two mt-haplotypes within the MK population. The light purple line represents 9,440 non-outlier SNP F_ST_ values between the two mt-haplotypes within the MK population. Two curves compare the MK population with a separate Rutgers’ population for the same 349 outlier SNPs: red is for individuals from the MK population with the southern mt-haplotype compared to the Rutgers population, and blue is for individuals from the MK population with the northern mt-haplotype compared to the Rutgers population.

To understand the magnitude of the 349 outlier *w*F_ST_ values, we compared these within population fixation index values to those between MK and a population 40 miles further south (Rutgers, NJ). Unexpectedly, MK *w*F_ST_ values were larger than the between population F_ST_ values (Fig 4). Rutgers only has the southern mt-haplotype, and between population F_ST_ values are a function of mt-haplotype: F_ST_ values are smaller for comparisons of nuclear SNPs between Rutgers and just individuals in MK with southern mt-haplotypes than for comparisons of Rutgers to individuals in MK with northern mt-haplotypes (Fig 4, red *versus* blue curve). Specifically, comparisons using MK individuals with northern mt-haplotypes are right-shifted with more loci with large F_ST_ values. These data indicate that the genetic distances of the 349 outlier SNPs between mt-haplotypes are larger within the MK populations than between populations and that the genetic distances between populations for nuclear SNPs are a function of the mt-haplotype.

In summary, the MK population is a well-mixed population with a few hundred unlinked nuclear SNPs that have significant allele frequency differences between the two mt-haplotypes (Fig 1). These biased allele frequencies create large *w*F_ST_ values that are distinct from the rest of the genome and unlikely to occur based on data permutations. The hypothesis that the MK population is well-mixed is supported by **A)** Admixture analysis on 3-population 3,700 nuclear SNPs (Fig 2), **B)** the DAPC indicating a single population based on all 11K nuclear SNPs (S6 Fig) and **C)** the few significant SNPs in LD across the genome or with the mt-haplotype (S3 and S4 Figs). These data, and the observation that MK *w*F_ST_ values among 349 outlier nuclear SNPs are larger than the F_ST_ value for the same loci among populations (Fig 4), suggest that that demographic effects including migration would not cause an association between the mitochondrial and nuclear genomes. These 349 outlier nuclear SNPs have an allele frequency difference between mt-haplotypes of 11.19% (95% CI = 10.69 to 11.69%), more than 3 times larger than the 3.28% (95% CI = 3.22% to 3.35%) allele frequency difference for the remaining 9,440 non-outlier, non-significant SNPs (S1 Fig). For the 349 outlier SNPs, this allele frequency difference translates to *w*F_ST_ values >0.054 (Fig 1), compared to the majority of SNPs (9,440, 81%) where 95% of *w*F_ST_ values are <-0.001 and have p-values >0.1 (Fig 1). The evolutionary importance of these 349 outlier SNPs is suggested by *w*F_ST_ values that are not likely to occur relative to other SNPs that share similar He. These differences are not due to different allele frequencies (MiAF = 0.132 for the 349 outlier *versus* 0.162 for 9,440 remaining SNPs, S1 Fig) or heterozygosity (0.23 and 0.20 for 349 outlier and 9,440 non-significant SNPs, respectively). Yet for the 349 outlier SNPs, as indicated by *w*F_ST_ values, the allele frequency differences between the two mt-haplotypes are significantly larger than the allele frequency variance within these groups; this is unusual relative to 96% of the other SNPs. These data on the 349 outlier nuclear SNPs are surprising given what we know about *F*. *heteroclitus* ecology and reproduction: individuals occupy small home ranges in estuaries [36, 72] and share a common reproductive strategy of laying and fertilizing eggs in the upper intertidal zone [73, 74]. We tentatively conclude that these 349 outlier SNPs are most likely evolving by natural selection due to the interactions between the nuclear and mitochondrial genomes.

### OxPhos function

**Fig 5:**
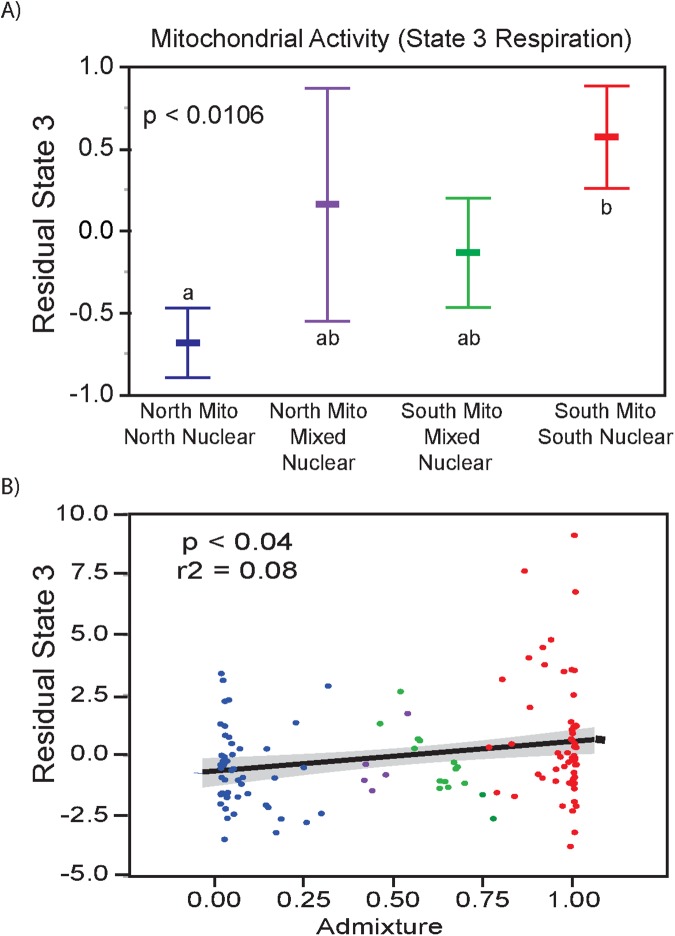
Mito-nuclear effects on State 3 Respiration. State 3 OxPhos metabolism in the MK population is oxygen respiration dependent on substrates and ADP, and the data graphed are the residuals from Admixture ancestry, body mass, acclimation temperature and assay temperature [109]. **A:** ANCOVA (p-val = 0.0194): individuals were assigned to one of four groups based on their 34 outlier genotypes and mt-haplotypes: ‘North Mito North Nuclear’ are individuals with >70% of alleles from the 349 outlier SNPs associated with northern mt-haplotypes, “South Mito South Nuclear” with >70% of alleles from the 349 outlier SNPs associated with southern mt-haplotypes, and the two mixed ancestral groups: individuals where >30% of nuclear genotypes are associated with the opposite mt-haplotype, as defined by STRUCTURE. Means (dot) and standard errors are displayed. N = 155. Letters (“a”, “b” are significantly different based on Tukey post-hoc comparison). **B:** Regression of residuals of State 3 *versus* fraction of southern ancestory (0.0–1.0) as defined by STRUCTURE with K = 2. Linear regression is significant (p <0.0055) with an R^2^ = 0.062

If these differences in 349 outlier SNPs are meaningful, we would expect differences in biological functions between mito-nuclear genotypes. To determine if the 394 outlier SNPs affect a biological function, cardiac OxPhos metabolism was measured as State 3 respiration (an integrative measure of ADP and substrate dependent mitochondrial respiration) and compared to mito-nuclear genotypes among the MK individuals (Fig 5). In ANCOVA (with Admixture coefficients, acclimation, assay temperature and body mass as covariates), we used four mitochondrial groups as the main effect (Fig 5A). The four mitochondrial groups are based on STRUCTURE analysis of the 349 outlier SNPs (Fig 3C). Most individuals have >70% of nuclear alleles associated with one of the two mt-haplotypes (Fig 3C). However, 21 individuals have mixed ancestry; these individuals shared at least 30% of nuclear alleles with the opposite cluster (northern 349 SNP alleles with southern mt-haplotype or southern 349 SNP alleles with northern mt-haplotype). This defines four groups: the two main structure groups (Fig 3C) and two groups with mixed ancestry from each cluster (individuals with >30% of the alternative allele). Among these four mito-nuclear groups, State 3 is significantly different (Fig 5A, p < 0.0194); admixture was not significant (p >0.8) while mass, acclimation and acute temperature were significant (p < 0.05). State 3 respiration was significantly lower in individuals with the northern mt-haplotype compared to those with the southern mt-haplotype (Fig 5A, Tukey post hoc test). Individuals with “mixed” nuclear backgrounds showed intermediate mitochondrial respiration. For mixed ancestry individuals with a northern mt-haplotype, having a larger number of “southern” associated nuclear alleles increased respiration rates, whereas the opposite effect was observed for individuals with a southern mt-haplotype with a larger number of “northern” associated nuclear alleles. Thus, this analysis indicates that variation in the nuclear genome modulates mt-haplotype effects on OxPhos metabolism.

A second analysis regresses State 3 respiration against the fraction of southern mt-ancestry (Fig 5B) using the same covariates as above (Admixture coefficients, body, mass, acute and acclimation temperature). The fraction of southern mt-ancestry is significant (p < 0.0055) and explains 6% of the variance (Fig 5B). These data indicate that individuals with the greater number of southern alleles with a northern mt-haplotype have greater OxPhos metabolism and individuals with more northern alleles with a southern mt-haplotype have lower OxPhos metabolism. Notice, that we form a polygenic score for each individual using the 349 outlier SNPs, which defines the fraction of ancestry related to southern mt-haplotype. Thus, because the MK 349 SNPs have significantly biased allele frequencies and their inferred ancestry of K = 2 reflects the two mt-haplotype, significant regression indicates a significant mito-nuclear interaction.

The potential genes that could affect State 3 mitochondrial respiration include approximately 89 nuclear and mitochondrial proteins that form the five OxPhos complexes, and approximately 1,500 other nuclear genes involved in mitochondrial functions [75]. Using BLAST [76], we aligned the 64bp sequences containing outlier SNPs against the *F*. *heteroclitus* genome. Sixty-four base pairs were used because this is the length of the sequences retained from sequencing and pipeline analysis[77]. Although all outlier sequences aligned to the genome, only 162 aligned to annotated genes. Many of these genes encode transcription factors (*e*.*g*., zinc finger proteins), are involved in signaling pathways (*e*.*g*., 1-phosphatidylinositol 4,5-bisphosphate, GTPase or receptors), or are trans-membrane proteins. Four genes were identified that likely influence OxPhos metabolism (Table 1). The first, acyl-coenzyme A thioesterase, which is mitochondrially localized, affects intermediates in the citric acid cycle, which forms OxPhos substrates *via* lipid metabolism regulation [78–80]. A second gene, adenylate kinase, regulates mitochondrial respiration by altering ADP/ATP ratios [81] and creating feedback signal communication. A third gene, NAD-dependent malic enzyme, catalyzes a reaction that forms pyruvate from malate with the reduction of NAD+ to NADH [82–84]. The mitochondrial variant acts as a regulatory enzyme, allosterically activated by fumarate and inhibited by ATP [85]. These substrates, products and allosteric regulators are all involved in OxPhos metabolism. The fourth gene, ribosomal mitochondrial protein (MRP) S35, is responsible for translating the 13 mitochondrial proteins making up the OxPhos pathway. MRPs are linked to human mitochondrial disorders such as Leigh Syndrome, multiple mitochondrial dysfunctions syndrome, Russell-Silver syndrome, Spinocerebellar ataxia with blindness and deafness, Stuve-Wiedemann syndrome, and Usher syndrome [86].

**Table 1:** Genes of interest that aligned to 64bp sequences.

Scaffold	Position	Gene	e-value
191	383976	Acyl-coenzyme A thioesterase 9, mitochondrial	7E-16
9927	383976	Adenylate kinase isoenzyme	1E-23
9976	1473252	NAD-dependent malic enzyme, mitochondrial	5E-26
98	588044	Ribosomal mitochondrial protein S35	2E-19

The described genes may play an important metabolic role through mitochondrial OxPhos protein regulation and translation. Surprisingly, none of the 349 outlier sequences aligned to the 76 nuclear encoded proteins that form the OxPhos complexes. There are two possible reasons for this: 1) none of the 11,000 SNP sequences include OxPhos proteins, or 2) SNPs in these proteins are not affected by the mito-nuclear interactions. Five of the 11,000 SNPs mapped to nuclear proteins that directly participate in OxPhos: one in Complex I, two in Complex II, and two in Complex V. Yet, there was no allele frequency bias in these OxPhos proteins. Mito-nuclear interactions may still affect other nuclear OxPhos genes that were not sequenced in our study. However, given our data, we postulate that epistatic selection affects OxPhos functions and is acting upstream of the OxPhos pathway.

We can compare our SNP data to previously published gene expression data on mt-haplotype effects in *F*. *heteroclitus*. Flight, *et al*. [87] published the effects of mt-haplotype, sex and hypoxia on mRNA expression measured in individuals from the same population. We used their published microarray probes to determine the co-localization of our 349 significant outlier SNPs with the oligos used to measure gene expression. Among the 349 significant outlier SNPs, 136 SNPs are within 150Kb of the oligos used by Flight et al. [87] (S3 Table). For these 136 genes, 24 (18%) have a significant p-value (p <0.01) for one of the three main effects: sex, hypoxia or mt-haplotype. One SNP (S10023_121524) had a significant mt-haplotype effect and was ~55Kb distant from a gene annotated by Flight *et al*., as spectrin beta chain, brain 1. For the four genes listed in Table 1, two co-localized with oligos used by Flight. The SNP associated with acyl-coenzyme A thioesterase 9 is ~12Kb downstream from Flight’s diamine acetyltransferase 1 gene, which has a significant hypoxia effect. The SNP associated with adenylate kinase has the same annotation as in Flight’s microarray, is 32 Kb downstream, and is different between sexes. The significant SNPs are from sequences that cover less than 0.0001% of the genome, and Flight’s microarray investigated ~4,000 genes (~14% of *F*. *heteroclitus* annotated genes) [88]. The observation that any of our SNPs are co-localized to any of these support the concept that they are functionally and evolutionarily important.

Although significant associations between nuclear loci and mt-haplotype point towards selection on mito-nuclear interactions, an alternative explanation could be assortative mating. Allelic bias may occur if individuals can recognize mates with similar mt-haplotypes and accordingly, preferentially mate. However, no evidence of this has been documented in *F*. *heteroclitus*. Our data is more likely explained by selection on mito-nuclear interactions for two reasons. First, there is a large overlap in 349 significant loci found by using two different methods: Arlequin [57] F_ST_ test and Fisher Exact test. It is very unlikely that these significant associations are random. Second, Admixture using 3,700 SNP from the 3-population set (Fig 2) and DAPC using all 11K SNP from the MK SNP set (S6 Fig) indicate little if any population structure within the MK population. Assortative mating would have to be highly selective to maintain allelic bias because the unlinked 349 outlier SNPs would come to equilibrium if only drift and incomplete isolation was responsible. Thus, assortative mating seems unlikely; instead MK seems to be a well-mixed random breeding population.

### Maintenance of selectively important mito-nuclear loci

Difficult questions to answer are how two mt-haplotypes are maintained in a single population and how so many loci are potentially affected by natural selection due to GxG interactions. Theoretically, it is difficult to maintain functionally different mitochondrial haplotypes in a single population due to GxG interactions [54–56]. Mito-nuclear polymorphisms can be maintained with sex-linked loci under restricted conditions [56, 89]. Yet, the 349 outlier *w*F_ST_ loci are distributed over 100s of scaffolds, and thus it seems unlikely that they are limited to sex-linked chromosomes. Mutation-selection balance also seems unlikely because there is a high frequency of the minor alleles: the average heterozygosity for the 349 outlier *w*F_ST_ loci is 0.23 and is similar to neutral loci. It is also difficult to suggest migration or other demographic effects because the allele frequency difference within MK population for the 349 outlier *w*F_ST_ loci is larger than the allele frequency difference among populations (Fig 4). Additionally, the 3-population data set indicates a well-mixed population (Fig 2). The high allele frequencies for both alleles among the 349 outlier *w*F_ST_ loci (S1 Fig) suggest that there is balancing selection, which might arise if the two alleles have different fitness effects in different environments. GxE interactions where allele effects have a high variance among environments could maintain selectively important polymorphisms especially if there is extensive pleiotropy [56] or unpredictable environmental variations [90]. Notice, because these individuals were captured together in the same estuarine creek, it is unreasonable to suggest spatial variation in the environment; instead temporal variation is common in the *F*. *heteroclitus* environment and may contribute to maintaining the observed mito-nuclear genetic variation.

What we do know is that there is environmentally dependent, adaptive divergence in OxPhos mRNA expression among populations [39, 91–93], suggesting that GxE interactions are possible. In other species, mito-nuclear interactions have pleiotropic effects [11, 22] and affect genome wide mRNA expression patterns [94, 95]. Thus, although lacking data to specifically address the evolutionary genetics that maintain selectively different mito-nuclear interactions, we suggest that temporal environmental variation affects mito-nuclear polymorphisms that have pleiotropic effects. The hypothesis of pleiotropic effects is supported by the diversity of annotations associated with the 349 outlier *w*F_ST_, which include transcription factors and signaling pathway genes that are likely to have a wide diversity of phenotypic effects.

## Conclusion

Although epistatic interactions between mitochondrial and nuclear genes have been shown to affect overall organismal fitness and metabolic activity [5, 8, 11, 14, 15, 22–24, 96], these studies have used divergent mt-haplotypes and divergent nuclear backgrounds or fail to show an effect on allele frequencies in natural populations. The data we present show that mito-nuclear interactions influence allele frequencies in a natural, freely interbreeding population. We show that 349 outlier SNPs have greater allele frequency differences between mt-haplotypes than within a mt-haplotype, creating large, significant *w*F_ST_ values (*w*F_ST_ values are F_ST_ values within a population between two mt-haplotypes). The distribution of *w*F_ST_ values within and F_ST_ values between populations for neutral SNPs is different from that of the 349 outlier SNPs. These 349 outlier SNPs were used to form a polygenic factor, where the individual scores affected OxPhos metabolism, supporting the hypothesis that mito-nuclear interactions are evolutionarily important. These observations are difficult to resolve with any neutral or realistic demographic mechanisms. Thus, we tentatively conclude that the most parsimonious explanation is that selection on mito-nuclear interactions is strong enough to alter allele frequencies for 100s of SNPs. The observation that several of these genomic SNPs are for genes that modulate OxPhos supports this hypothesis. The selection for mito-nuclear interactions that modulate OxPhos may occur if there is extensive standing genetic variation and the genes have small effects.

## Materials and methods

### Datasets

For the sake of clarity, we use two informative names for the two datasets analyzed in this manuscript. “MK-specific” refers to the SNP dataset that is solely based on the 180 individuals from New Jersey. To define admixture, a second data set (“3-population”) includes these MK individuals and individuals from Maine (ME, n = 35) and Georgia (GA, n = 38). SNP discovery pipeline [77] defines SNPs that are polymorphic with specific frequency and read depth, and thus, while many of the SNPs in the MK-specific and 3-population dataset are the same, 46% are unique.

### Experimental animals

Adult *F*. *heteroclitus* were collected during the summer months from Mantoloking, NJ (40.049427°N, -74.065087°W), Wiscassett, ME (43° 57’ 15.10”N, 69° 43’ 13.64”W), and Sapelo Island, GA (31° 27’ 13.39”N 81° 21’47.65”W). All fish were captured in minnow traps with little stress and removed in less than one hour. Fieldwork was completed within publicly available lands, and no permission was required for access. *F*. *heteroclitus* does not have endangered or protected status, and small marine minnows do not require collecting permits for non-commercial purposes. All fish were acclimated for 4 weeks to either 12°C or 28°C, temperatures naturally encountered in their natural environment. These two acclimation temperatures were used to explore how chronic (acclimation) and acute temperatures affect physiological functions [97]. Fish were exposed to a 14 hour light cycle, kept at 15ppt salinity and fed twice a day, 7 days a week. Housing, acclimation and non-surgical tissue sampling protocols were in compliance with and approved by the University of Miami Institutional Animal Care and Use Committee (IACUC).

### Isolating DNA

DNA was isolated from fin clips and stored in 270 ul of Chaos (buffer 4.5M guanidinium thiocynate, 2% N-lauroylsarcosine, 50mM EDTA, 25mM Tris-HCL pH 7.5, 0.2% antifoam, 0.1M β-mercaptoethanol) with ~ 1g of silica beads and combined with 130 ul of 10X TE (100mM Tris pH 7.8, 10mM EDTA pH 8.0). Tissue was homogenized using zirconium beads. Supernatant was removed and placed in a new tube with 200 ul of 95% EtOH and mixed. This solution was then quickly added to silica columns for DNA isolation. Loaded columns were centrifuged for 1 minute at 6,000xg, and flow through was discarded. As modified from [98], columns were washed three times with 750 ul of protein wash buffer (70 ml 96% EtOH and 26 ml binding buffer which contained 6M guanidine thiocyanate, 20 mM EDTA pH 8.0, 10 mM Tris-HCl pH 7.5, 4% Triton X-100) followed by centrifugation for 1 minute at 6,000xg. Then, samples were washed with 650 ul wash buffer (60% EtOH, 50mM NaCl, 10mM Tris-HCl pH 7.4, 0.5mM EDTA pH 8.0) and centrifuged for 1 minute at 16,100xg followed by another wash with 650 ul wash buffer and centrifugation for 3 minutes at 16,100xg to dry the silica column. 100 ul of 0.1XTE (10mM Tris, 0.1 mM EDTA) was added to elute the genomic DNA upon centrifugation for 1 minute at 6,000xg.

### Genotyping mitochondria

Mitochondrial haplotypes were defined by PleI, and BstYI restriction digest of ND2 and cytochrome b respectively. The digests were run on a 1% agarose gel to separate DNA fragments. Individuals from Maine and Georgia were used as controls. Both restriction enzymes yielded the same results for each individual. Haplotypes defined by restriction enzymes were the same as mitochondrial SNPs identified by GBS (genotyping by sequencing [50]). There were 19 mitochondrial SNPs that were in complete linkage disequilibrium, LD, (D’ 1.0). A single mitochondrial SNP was imputed for all individuals and used to determine relationships among nuclear-mitochondrial genotypes.

### Genotyping by sequencing

Isolated DNA quality was assessed via gel electrophoresis, and concentrations were quantified using Biotium AccuBlue High Sensitivity dsDNA Quantitative Solution according to manufacturer’s instructions. After quantification, 100 ng of DNA from each sample was dried down in a 96-well plate. Samples were then hydrated overnight with 5 ul of water before AseI restriction enzyme digestion. This digest, based on *in silco* digest of the *F*. *heteroclitus* genome (NCBI accession JXMV00000000.1 [99]), should produce 523,349 fragments with 117,639 <500bp in size. Adaptors with separate barcodes for each individual (0.4 pmol/sample) were ligated to the genomic DNA after digestion with AseI. DNA samples were then pooled and purified using an equal volume of carboxyl coated magnetic beads (Fisher Scientific) in a PEG/salt solution (0.5 g beads in 100 mls of 20% PEG 8000, 2.5 M NaCl). Two bead purifications were used to select fragments between 100 and 400 bp. First, DNA less than 400 bp was separated from larger DNA which is bound to magnetic beads at low NaCl_2_ concentration (0.87 M), then bead-salt solution was raised (NaCl_2_ at 1.25M) so that only DNA larger than 100 bp are bound. These beads were washed with 70% EtOH, and DNA was eluted. The size range of purified products was verified using Agilent 2100 Bioanalyzer (Santa Clara, CA). A range of PCR cycles on the 100-400bp genomic fragments was used to optimize the amplification of restriction fragments using primers that anneal to the adapters. The distribution and concentration of the amplified library was verified using Agilent 2100 Bioanalyzer (Santa Clara, CA). DNA from the 18-cycle run formed the GBS library that was sequenced (Illumina Hi Seq 2500, 75bp single end reads; Elim Biopharmaceuticals, Inc., Hayward, CA).

### GBS bioinformatics

The Java program, TASSEL [77] used the first 64bps of single end sequences and aligned them to the *F*. *heteroclitus* genome to call SNPs. The *F*. *heteroclitus* genome (NCBI accession JXMV00000000.1), which consists of 10,180 scaffolds plus mitochondria, was used to map sequencing reads. Two GBS datasets were produced: 1) the MK-specific, and 2) the 3-population dataset. For the MK-specific dataset, individuals were removed that had less than 50% of SNPs, reducing the number of individuals from 180 to 155. The data were filtered to remove SNPs with less than 1% minimum allele frequencies that occurred in less than 70% of individuals. Hardy-Weinberg expectation was calculated for individual loci using Arlequin v3.5.1.2 [57], and we excluded 256 SNPs where Ho>He and was significant (p<0.01). This latter filter is used to remove potential SNPs that represent differences between paralogs *versus* true allelic variants for a single locus [100]. For the 3-population dataset, individuals were removed that had less than 30% of SNPs, reducing the number of individuals from 257 to 234. SNPs that occurred in less than 77% of individuals were removed.

### Population analyses

For the MK-specific dataset, allele frequencies were defined using adegenet in R [68], and minor alleles were defined among all 155 NJ individuals. That is, a minor allele was defined across all individuals even when their frequencies >0.5 within a mt-haplotype. Two approaches were used to identify allele frequencies that had a bias relative to mt-haplotype: Fisher-Exact test and outlier-test using Arlequin v3.5.1.2 [57]. The Fisher-Exact test determines the bias in allele frequencies at each locus relative to mt-haplotype using PLINK [101]. Arlequin was used to compare the relative genetic distance between the two mt-haplotype relative to other loci. Specifically, we used an outlier test to define fixation index (F_ST_) values that exceed the expectation based on the observed data. For comparisons between the two mt-haplotypes, we use fixation index (F_ST_), and for clarity we use *w*F_ST_ (within population among mt-haplotypes). To identify SNPs with *w*F_ST_ outlier values, we used Arlequin v3.5.1.2 [57]. Outlier *w*F_ST_ values are based on FDIST [58, 102] as implemented in Arlequin, where coalescent simulations are used to get a null distribution and confidence intervals around the observed values and then tested to determine if observed locus-specific *w*F_ST_ values can be considered as outliers conditioned on the globally observed *w*F_ST_ value.

For Admixture analysis we thinned the 3-population dataset, removing SNPs closer than 100 bp (as suggested by the Admixture manual [103]). Thinning resulted in 3,700 SNPs. These 3,700 SNPs were input into Admixture v.1.3.0 [104] to infer ancestries of ME, GA, and MK individuals and provide an unbiased estimation of overall population structure.

LD was determined for MK individuals in 1) all SNPs in the MK-specific dataset, and 2) among 3.7K thinned SNPs from the 3-population dataset [77]. LDs were determined using a moving 50bp-SNP window providing r^2^ (correlation coefficient), D’ and p-values associated with pairs of SNPs within and among scaffolds. The significant LD between SNP pairs and each SNP with mt-SNPs are reported as p-values <0.01 and with FDR correction [105]. FDR based on Benjamini & Hochberg [105] and were calculated in R using p.adjust [106].

Tajima’s D was calculated using VCFtools [66] with 50bp non-overlapping windows. VCFtools uses the physical distance (50bp) to calculate Tajima’s D. We used a 50bp window because nearly all SNPs within a 64 bp tag are captured by this window (i.e. SNPs occur at +10 bp in a tag-sequence). Using a 100bp window produced nearly identical results.

STRUCTURE v2.3.4 [69] was used to identify the number of ancestral populations (K) with similar allele frequencies and was also used to predict the magnitude of admixture within the single collection site. CLUMPAK [107] was used to average output from multiple STRUCTURE runs. For the 349 outlier SNPs, models allowing admixture and correlated gene frequencies were used with seven independent runs for each K-value from 1–5. Eleven thousand permutations with 11,121 initial runs (burn-in) were used for each run. The most parsimonious K was defined as that with the most likely K (largest mean Ln-likelihood) and the ΔK was based on the rate of change Ln-likelihood [70] using STRUCTURE HARVESTER [71]. We chose the most likely K if K was equal to 1, and used ΔK for K where the most likely was greater than one because ΔK can only resolve the best K with K >1.

Discrimination analysis of principal components (DAPC) was conducted in R using ‘adegenet’ [68]. DAPC uses the principal components of allele frequencies to infer the number of clusters of genetically related individuals by partitioning into a between-group and within- group component and maximizing discrimination between groups [108].

### Co-localization of SNPs and microarray data

To compare SNP genome locations (position on specific scaffold) to the location of oligo-nucleotides used by Flight, *et al*. [87] in the construction of their microarray, we use bwa to align the oligo-nucleotides to the most recent *F*. *heteroclitus* genome at NCBI (GCA_000826765.1 Fundulus_heteroclitus-3.0.2 scaffolds). Most oligos used in their microarray are >250bp and few full-length oligos aligned to the genome (most likely due to introns). To overcome this problem, we used non-overlapping 50bp windows for the alignments.

### OxPhos metabolism

All individuals used for GBS analyses had their cardiac OxPhos metabolism measured as described in [109, 110]. Heart ventricles were dissected, cut into halves, and half was placed into a muscle relaxation solution (10 mM Ca-EGTA buffer, 0.1 µM free calcium, 20 mM imidazole, 20 mM taurine, 50 mM K-MES, 0.5 mM DTT, 6.56 mM MgCl2, 5.77 mM ATP, 15 mM phosphocreatine, pH 7.1) [111]. The other half was saved for future RNA work. Tissues were then permeabilized using 2.5mg/ml saponin solution for 15 minutes, followed by 4 washes in relaxation solution for 5 minutes each [111]. Once permeabilized, tissues were immediately transferred to the respirometry chambers containing a respiration medium (5mM EGTA, 3mM MgCl2.6H2O, 60mM K-lactobionate 20mM Taurine, 10mM KH2PO4, 20mM HEPES, 110mM Sucrose, 1g/l BSA).

The acute effect of temperature on mitochondrial activity was measured at three temperatures: 12°C, 20°C, and 28°C. Activity was measured and analyzed using the Oxygraph 2-k and DatLab software (OROBOROS INSTRUMENTS, Innsbruck, Austria). Population, acclimation temperature, and acute temperature changes were all randomized. All OxPhos determinations were relative to the amount of DNA in the measured tissue. Respiration rates were measured as pmol O_2_ s^-1^ ml^-1^ per ng DNA. The detailed analyses of acclimation and acute effect on OxPhos function within population are lengthy and are the subject of a separate publication [109].

After addition of the tissue to the respiration chamber, state 3 was determined. State 3 is defined as routine oxygen consumption resulting in ATP production in the presence of substrates and ADP. First, the substrates pyruvate (5 mM), glutamate (10mM), and succinate (10mM) were added, followed by ADP addition (5mM, state 3); cytochrome c (10μM) was added to check mitochondrial membrane integrity [111]. The tissue was recovered after respiration assays, and total DNA was quantified using AccuBlue high sensitivity dsDNA quantitation solution (Biotium). All activity was normalized by ng/ul of DNA. OxPhos function is represented as residuals from acclimation, assay temperature, body mass, and percent admixture from the 3-population SNP dataset. Percent admixture from Admixture v1.3.0 had no significant effect on OxPhos function [112].

### Ethics

Adult *F*. *heteroclitus* were captured in minnow traps with little stress and removed in less than 1 hour. Fieldwork was completed within publicly available lands, and no permission was required for access. Housing, acclimation and non-surgical tissue sampling protocols were in compliance with and approved by the University of Miami Institutional Animal Care and Use Committee (IACUC).

## Supporting information

S1 Table 11,705 nuclear loci specified as significant outliers or non-significant. (XLSX)

S2 Table Annotations, positions and variant types for 349 significant SNPs. (XLSX)

S3 Table Co-localization of 349 significant SNPs with Flight's gene expression data. (XLSX)

S1 Fig Differences in minor allele frequencies (MiAF). Differences in MiAF between northern and southern mt-haplotypes *versus* overall MiAF (minor allele frequencies among all individuals). Blue dots indicate values for the 349 outlier SNPs.
(TIF)

S2 Fig Ten million random F_ST_ values *versus* 349 outlier *w*F_ST_ values. One thousand random permutations of ten thousand SNPs with non-significant *w*F_ST_ values. *w*F_ST_ values were determined when individuals were randomly assigned to one of two groups at the same frequency as mt-haplotypes. Rarely were *w*F_ST_ values equal to or greater than the 349 outlier F_ST_ values.
(TIF)

S3 Fig LD for 3 Population 3,700 SNPs. Linkage disequilibrium for 3,700 SNPs among three populations: Maine, MK-NJ and Georgia. Plots are Log_10_ distance (bp) *versus* negative log_10_ for the FDR p-value (1 = 10% FDR). Distances are within scaffolds for 3,700 SNPs thinned so that all SNPs >100bp apart.
(TIF)

S4 Fig LD among MK 11K SNPs. SNPs from MK, New Jersey population **A**: Histogram of log_10_ distance in base pairs between SNPs for all 11K SNPs. **B:** Histogram of log_10_ distance in base pairs for SNPs with significant LDs (FDR 10%). 66 SNPs are in LD with another SNP > 100 bp away. **C:** Distribution of p-values for significant LD (FDR 10%) *versus* log_10_ distance in base pairs. Dark blue solid spots are for the 349 outlier SNPs.
(TIF)

S5 Fig Tajima’s D for Significant and Non-significant. The relative frequency (density) Tajma’s D based on 50 basepair windows that include the 349 outlier SNPs (blue) or only the non-significant SNP (red). Tajima D values were calculated using VCFtools.
(TIF)

S6 Fig DAPC for 11K SNPs from the MK dataset. A) Results of the Bayesian information criterion used to infer the number of genetic clusters. B) Discrimination of two mt-haplotypes based on all 11K SNPs. Discriminant function separates individuals into one group based on mt-haplotype. “North” is for northern mt-haplotype, “South” is for southern mt-haplotype.
(TIF)
