# Supplementary figures and images for: Evolved genetic and phenotypic differences due to mitochondrial-nuclear interactions
Source: PLoS Genet. 2017 Mar 31;13(3):e1006517. doi: 10.1371/journal.pgen.1006517 (PMC5375140; doi:10.1371/journal.pgen.1006517)

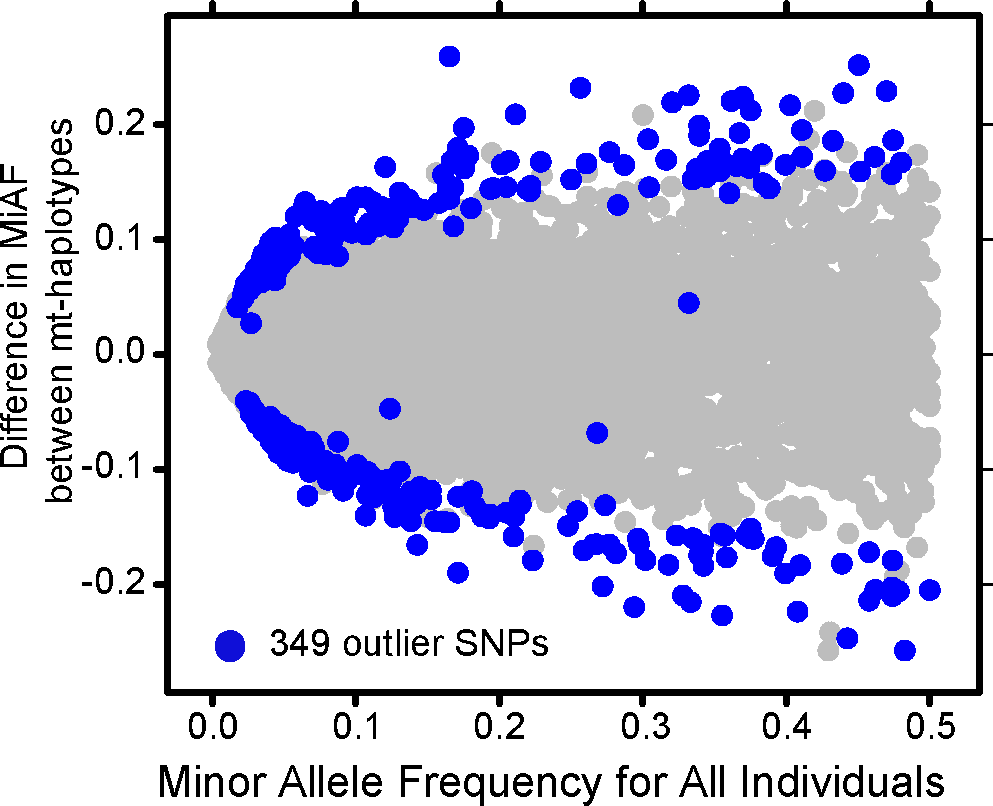

Supplement: S1 Fig — Differences in MiAF between northern and southern mt-haplotypes versus overall MiAF (minor allele frequencies among all individuals). Blue dots indicate values for the 349 outlier SNPs. (TIF) [file pgen.1006517.s004.tif]

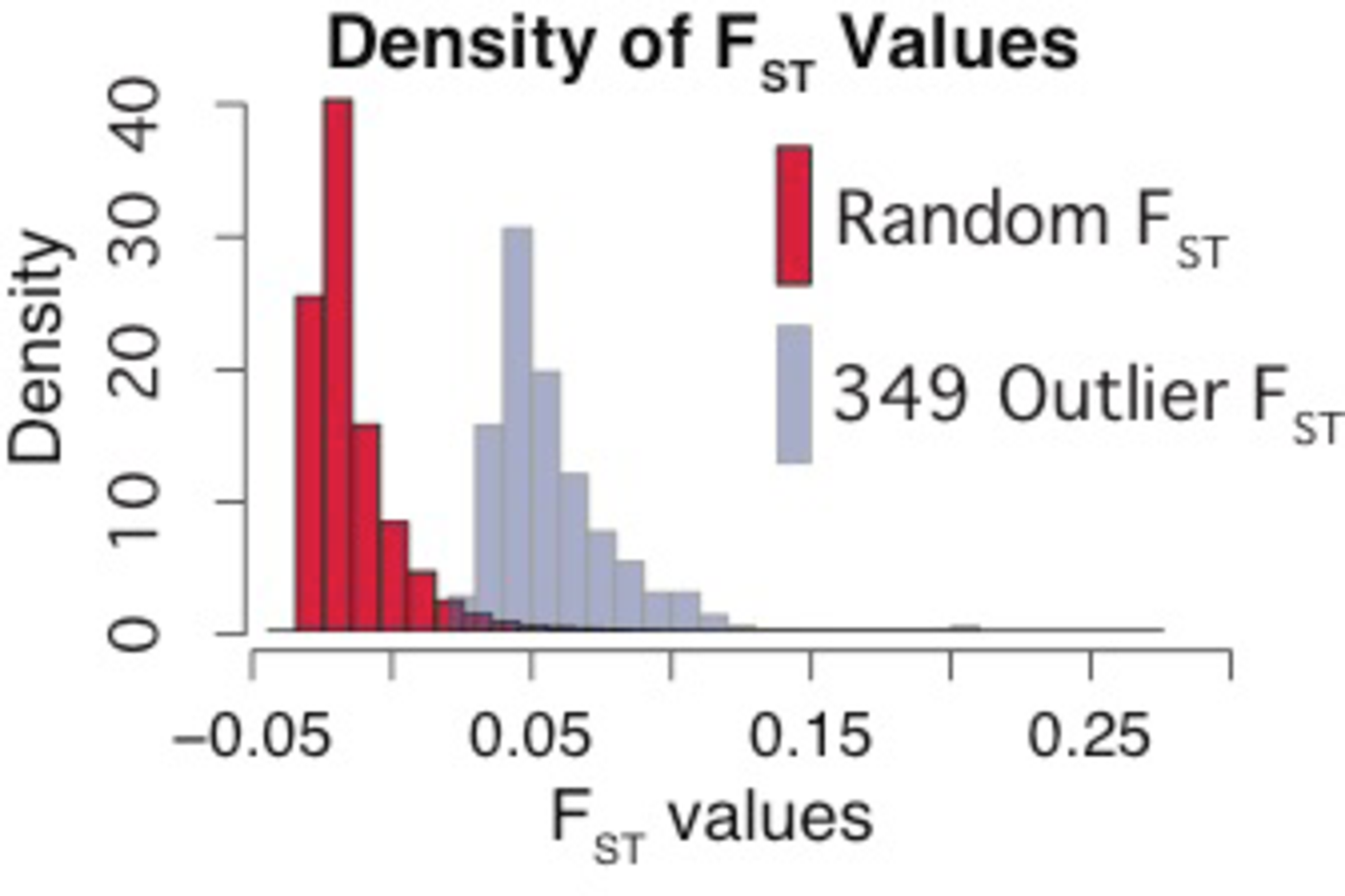

Supplement: S2 Fig — One thousand random permutations of ten thousand SNPs with non-significant wFST values. wFST values were determined when individuals were randomly assigned to one of two groups at the same frequency as mt-haplotypes. Rarely were wFST values equal to or greater than the 349 outlier FST values. (TIF) [file pgen.1006517.s005.tif]

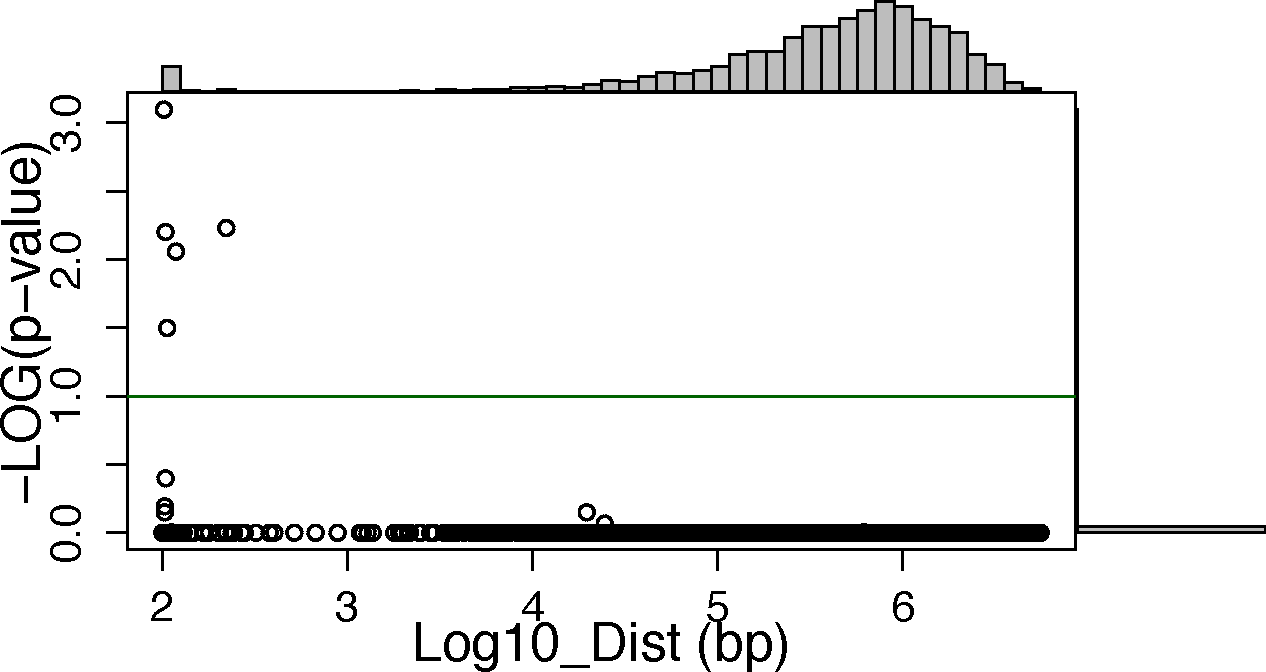

Supplement: S3 Fig — Linkage disequilibrium for 3,700 SNPs among three populations: Maine, MK-NJ and Georgia. Plots are Log10 distance (bp) versus negative log10 for the FDR p-value (1 = 10% FDR). Distances are within scaffolds for 3,700 SNPs thinned so that all SNPs >100bp apart. (TIF) [file pgen.1006517.s006.tif]

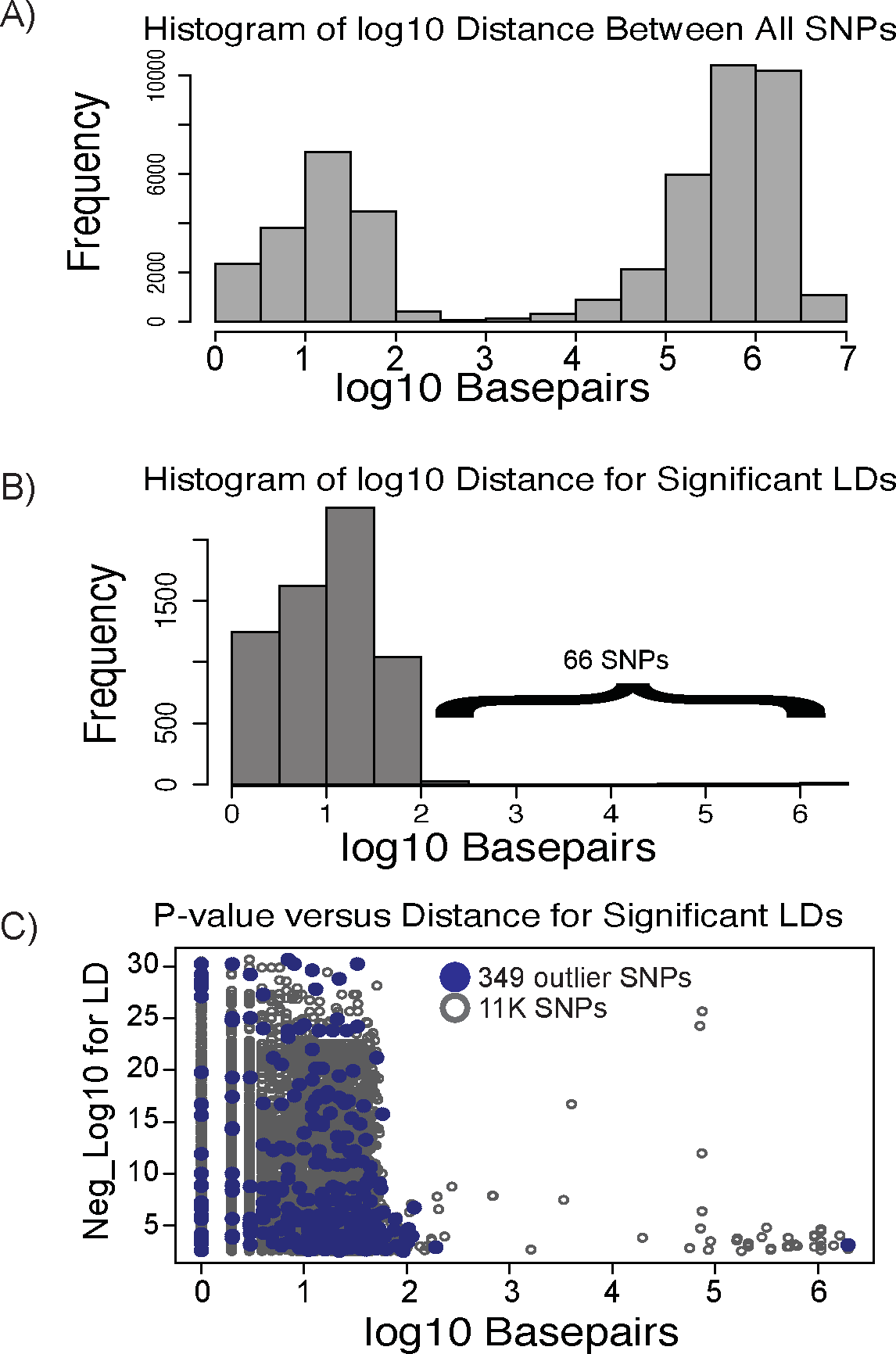

Supplement: S4 Fig — SNPs from MK, New Jersey population A: Histogram of log10 distance in base pairs between SNPs for all 11K SNPs. B: Histogram of log10 distance in base pairs for SNPs with significant LDs (FDR 10%). 66 SNPs are in LD with another SNP > 100 bp away. C: Distribution of p-values for significant LD (FDR 10%) versus log10 distance in base pairs. Dark blue solid spots are for the 349 outlier SNPs. (TIF) [file pgen.1006517.s007.tif]

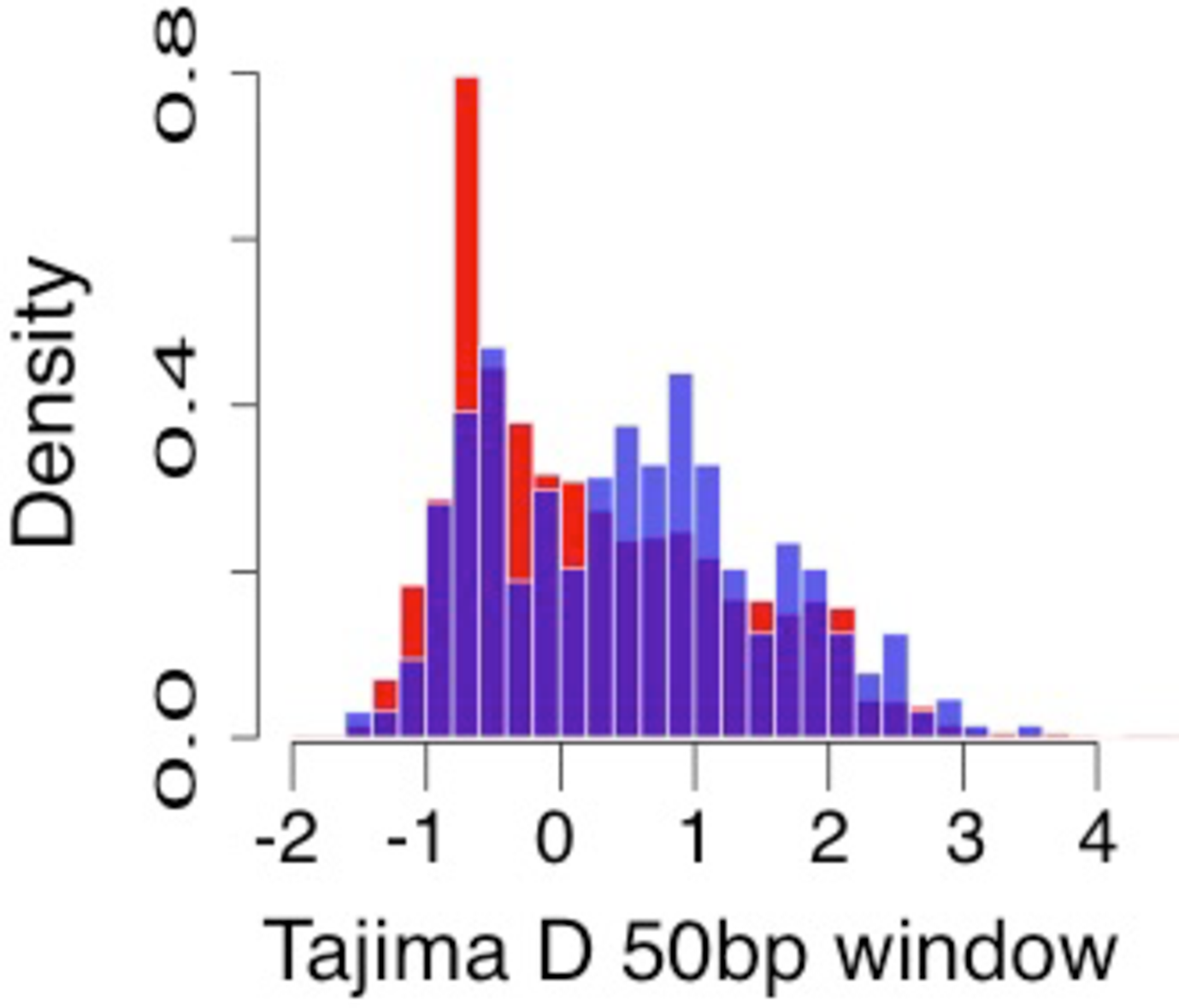

Supplement: S5 Fig — The relative frequency (density) Tajma’s D based on 50 basepair windows that include the 349 outlier SNPs (blue) or only the non-significant SNP (red). Tajima D values were calculated using VCFtools. (TIF) [file pgen.1006517.s008.tif]

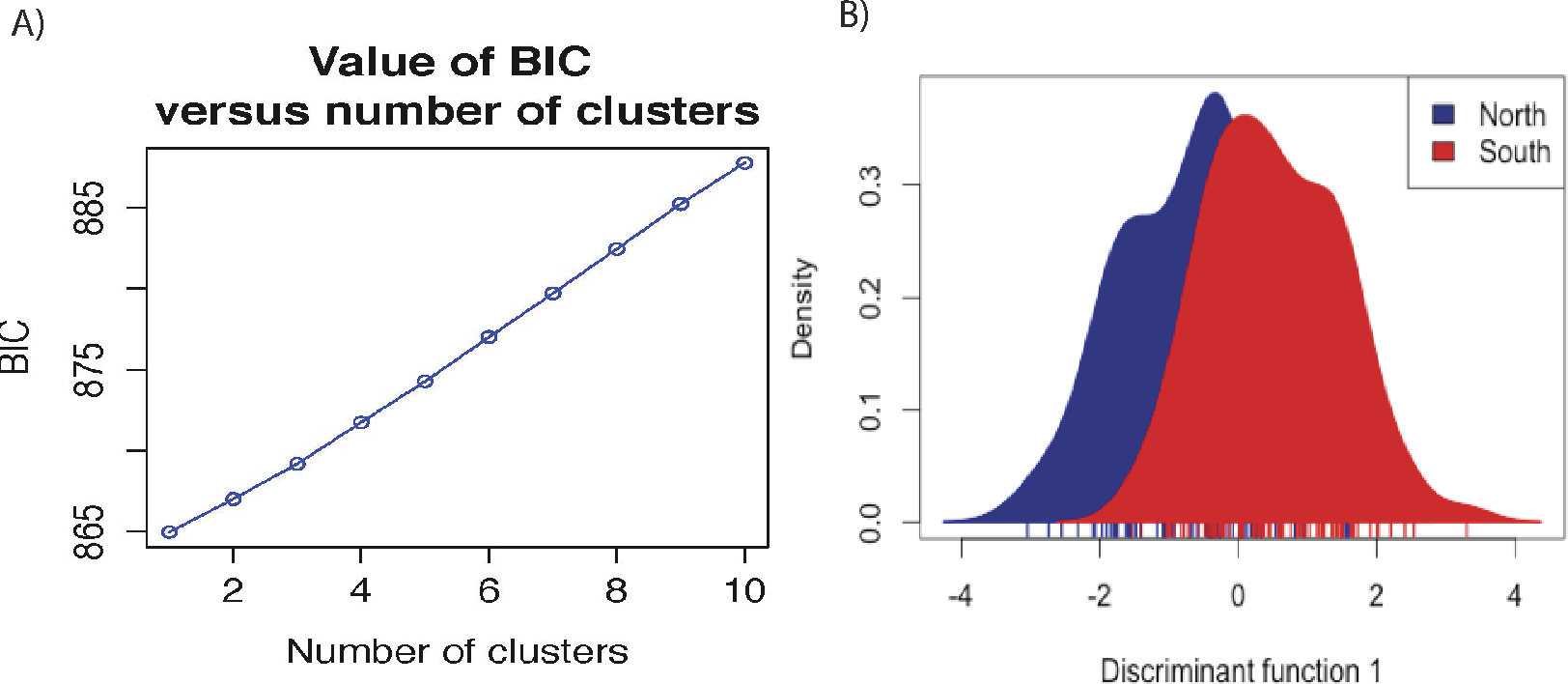

Supplement: S6 Fig — A) Results of the Bayesian information criterion used to infer the number of genetic clusters. B) Discrimination of two mt-haplotypes based on all 11K SNPs. Discriminant function separates individuals into one group based on mt-haplotype. “North” is for northern mt-haplotype, “South” is for southern mt-haplotype. (TIF) [file pgen.1006517.s009.tif]
